# Bilateral Anterior Uveitis in the Context of Concurrent HIV and Syphilis Co-infection

**DOI:** 10.7759/cureus.63024

**Published:** 2024-06-24

**Authors:** Devaun M Reid, Sunny Kahlon, Monica Khadka, Nishanth Chalasani, Thomas A Weppelmann

**Affiliations:** 1 Internal Medicine, University of South Florida Morsani College of Medicine, Tampa, USA; 2 Ophthalmology, Tampa General Hospital, University of South Florida, Tampa, USA

**Keywords:** ophthalmology, hiv care, syphilis uveitis, bilateral anterior uveitis, anterior uveitis

## Abstract

This case report presents bilateral anterior uveitis (BAU) in a 26-year-old male concurrently infected with HIV and syphilis, highlighting a rare and complex clinical presentation. BAU, typically linked with systemic diseases, poses significant diagnostic and therapeutic challenges when co-occurring with such infections. Despite common associations with posterior uveitis in co-infected individuals, this patient displayed BAU, underscoring the variability in ocular manifestations. The case details the clinical progression, diagnosis, and management strategies, emphasizing the need for comprehensive ophthalmologic and systemic evaluation. The report aims to enhance awareness and understanding of the implications of concurrent HIV and syphilis infections in ocular inflammation, advocating for tailored treatment approaches and a high index of suspicion in similar presentations.

## Introduction

Bilateral anterior uveitis (BAU) is an ocular inflammatory condition affecting the anterior chamber of both eyes, commonly associated with systemic diseases or infections. Previous literature has established associations between uveitis and HIV and *Treponema pallidum* infection (syphilis) individually [1]. As of 2022, the global prevalence of HIV stands at approximately 39 million, and syphilis, despite available treatments, continues to manifest in specific populations [2-3]. Co-infection of HIV and syphilis can potentiate the clinical manifestations and complications of each pathogen, and such an interaction may lead to a more pronounced ocular inflammatory response [4]. Existing case reports have documented anterior uveitis in patients infected with either HIV or syphilis, but BAU cases in the setting of concurrent HIV and syphilis infection remain underrepresented in the literature [5-6]. HIV and syphilis co-infection typically present with posterior uveitis, especially in patients with low CD4 counts [6]. In this case report, we detail the clinical findings of a patient diagnosed with BAU alongside HIV and syphilis co-infection. Our discussion encompasses clinical and ophthalmologic findings, systemic associations, and therapeutic strategies, aiming to enhance current knowledge on this specific clinical association.

## Case presentation

We present the case of a previously healthy 26-year-old male who initially presented with left eye pain and sensitivity to light that had persisted for several weeks. He was diagnosed with conjunctivitis and iritis and was prescribed antibiotics and cycloplegic drops, but these treatments did not alleviate his symptoms. His past medical history included no smoking, occasional alcohol consumption, and no preexisting medical conditions. Subsequently, he presented to the emergency department with bilateral eye pain, blurry vision, sensitivity to light, and dysuria. Physical examination revealed a fever of 100.5°F and bilateral eye redness. An ophthalmologic examination showed visual acuity of 20/50 in the left eye and 20/30 in the right eye, with normal intraocular pressure and pupils equal and reactive. Fundoscopic examination showed limited visualization of retinal vessels, and the patient was tearing bilaterally.

**Figure 1:**
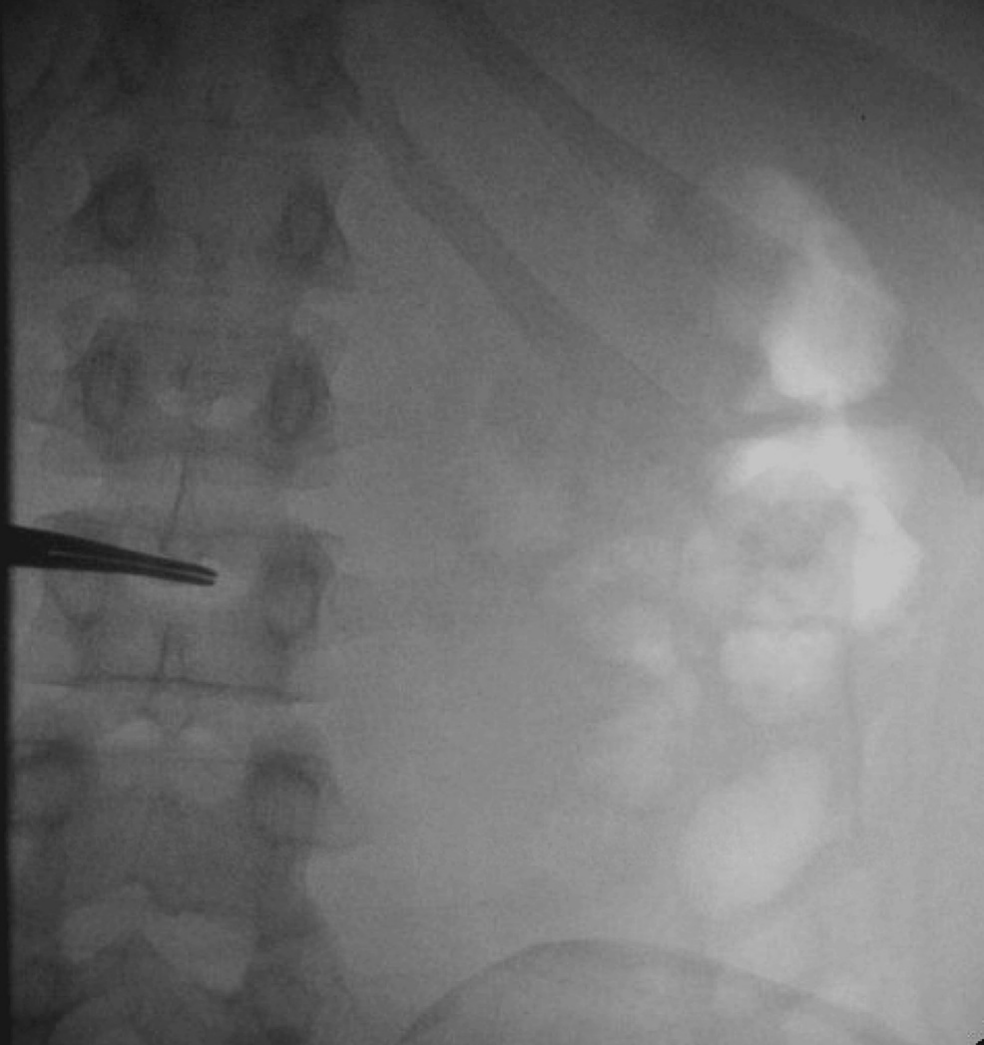
Fluoroscopically guided lumbar puncture

After the visit to the emergency room, he was given a cyclopentolate 0.5% and gonioscopic hypromellose 2.5% ophthalmic solution. Additionally, he was referred to ophthalmology for follow-up. One month later, he returned to the emergency department with complaints of burning left eye pain, photophobia, eye pain, and blurry vision. Examination revealed no foreign bodies, with both eyes showing corneal abrasion and fluorescein uptake. Laboratory results showed a WBC count of 3.57 K/uL, hemoglobin of 10 g/dl, and hematocrit of 31.3%, indicating normocytic anemia. Given the clinical presentation, anterior uveitis was suspected. Subsequent lab results returned positive for HIV, with a CD4 count of 54 and a viral load of 144,257 copies/mL. He was started on trimethoprim-sulfamethoxazole prophylaxis to prevent opportunistic infections. Treponemal antibody testing was reactive, raising suspicion of ocular syphilis. A lumbar puncture was performed, revealing an opening pressure of 19 cm H2O in the prone position, and 15 mL of clear cerebrospinal fluid (CSF) was obtained (Figure 1). The patient was treated with an intravenous penicillin-G regimen (4 million units IV every four hours).

During his hospital stay, his uveitis showed improvement with a change in eye drops from cycloplegic drops to steroid drops. He was also found to have an elevated RPR titer (1:128), further supporting the diagnosis of ocular/neurosyphilis. He reported the onset of symptoms in late December 2015, including left-eye light sensitivity, progressive blurry vision, and pain in eye movement. He experienced a 30-40 lb weight loss over four months, chills and sweats for one to two weeks, loose stools for one week two times a day, dysuria for one week, and right upper back pain for six days. He denied fevers at home, coughs, shortness of breath, chest pain, nausea/vomiting, new rashes, or sick contacts. He also denied any history of syphilis.

Upon diagnosis, he was treated with IV penicillin G (4 million units every four hours) for suspected neurosyphilis, trimethoprim-sulfamethoxazole for prophylaxis against opportunistic infections due to his low CD4 count, nystatin (500,000 units orally four times daily) for oral thrush, enoxaparin (40 mg subcutaneously daily) for deep venous thrombosis prophylaxis, and pantoprazole (40 mg orally or intravenously daily) for GI prophylaxis. Initially treated with cyclopentolate for his eyes, this was later changed to steroid drops as recommended by ophthalmology.

He was stabilized and discharged with a plan for outpatient follow-up, including the initiation of antiretroviral therapy (ART) and continued ophthalmology care. He was to follow up with the health department for ART initiation and with ophthalmology for ongoing eye care. His discharge diagnoses included BAU (likely ocular syphilis versus HIV reaction), newly diagnosed HIV/AIDS, syphilis (resolving), normocytic anemia, dysuria (resolved, likely due to syphilis), and an elevated gamma globulin gap (stable, secondary to HIV). He was educated about his diagnosis, treatment plan, and the importance of follow-up care to manage his HIV and associated conditions.

## Discussion

The uniqueness of our case lies in the presentation of BAU with concurrent HIV and syphilis co-infection, a clinical scenario scarcely reported in the literature. While anterior uveitis is a well-characterized ocular inflammation, its association with both HIV and syphilis in the same patient is rare, as reflected in our case.

The patient’s presentation with bilateral eye pain, photophobia, and blurry vision is typical of anterior uveitis, but the concurrent diagnosis of HIV and syphilis adds complexity to the case. Anterior uveitis often manifests with such symptoms, but the underlying etiologies vary widely [1]. Our patient’s low CD4 count and high HIV viral load are indicative of advanced HIV, which significantly increases the risk of ocular complications [4]. The coexistence of syphilis, another pathogen with known ocular manifestations, can increase the risk of clinical complications [7].

In cases of HIV and syphilis co-infection, posterior uveitis is more commonly reported [8]. However, our patient presented with BAU, diverging from this typical presentation. This distinction makes our case noteworthy, as it adds to the growing body of evidence that ocular manifestations in co-infected individuals can vary significantly. Another study highlights the importance of multimodal imaging in such cases, which could aid in better understanding and documenting the atypical presentations of ocular syphilis in HIV-co-infected patients [9].

Additionally, a previous discussion on the challenges in diagnosing ocular syphilis, especially in HIV-co-infected individuals, resonates with our case [10]. The empirical use of penicillin in our patient, despite a challenging diagnostic process, underscores the necessity of a high index of suspicion in similar clinical settings.

The resurgence of ocular syphilis is particularly relevant in urban and underserved communities [11]. While our case is not directly linked to these demographics, it aligns with the broader narrative of an increasing trend in ocular syphilis cases. This trend necessitates heightened vigilance and consideration of syphilis in differential diagnoses of ocular inflammations, especially in the context of HIV co-infection. Our case also draws a parallel to the study on the co-infection of parvovirus B19 and SARS-CoV-2, highlighting the complex interactions of multiple infections impacting ocular health [12-15]. While our case involves different pathogens, the underlying principle of co-infections leading to atypical presentations remains pertinent.

## Conclusions

Our case adds a unique dimension to the existing literature on BAU, particularly in the setting of HIV and syphilis co-infection. It underscores the need for a comprehensive evaluation and highlights the potential for atypical presentations in patients with multiple systemic infections. This case not only contributes to our understanding of the interplay between these infections and ocular health but also emphasizes the importance of considering a broad range of differential diagnoses in similar clinical scenarios.
